# Genetic modulation of soluble Aβ rescues cognitive and synaptic impairment in a mouse model of AD

**DOI:** 10.1186/1750-1326-8-S1-O30

**Published:** 2013-09-13

**Authors:** Stephanie Fowler, Angie Chiang, Ricky Savjani, Megan Larson, Dorothy Schuler, John Cirrito, Sylvain Lesne, Joanna Jankowsky

**Affiliations:** 1 Neuroscience, Baylor College of Medicine, Houston, TX, USA; 2 Texas A&M Health Science Center, College Station, TX, USA; 3 Neuroscience, University of Minnesota, Minneapolis, MN, USA; 4 N. Bud Grossman Center for Memory Research and Care, Institute for Translational Neuroscience, University of Minnesota, Minneapolis, MN, USA; 5 Neurology, Washington University School of Medicine, St. Louis, MO, USA; 6 Neurology, Neurosurgery, and Huffington Center on Aging, Baylor College of Medicine, Houston, TX, USA

## Background

There has been a longstanding debate on whether amyloid plaques are pathogenic by causing physical damage to surrounding tissue or protective by sequestering more toxic soluble forms of amyloid β (Aβ) peptide. Numerous studies in mouse models have documented a complex relationship between soluble Aβ, deposited aggregates, and cognitive decline, but few experimental systems have been capable of dissecting how each form of the peptide contributes to the progressive memory deficits of Alzheimer’s disease. Here we use a controllable transgenic model expressing a mutant form of amyloid precursor protein (APP) to distinguish the impact of APP and soluble Aβ from that of deposited amyloid on cognitive function and synaptic structure.

## Materials and methods

We created a transgenic model in which the tetracycline transactivator drives expression of APPswe/ind in neurons throughout the forebrain. After 6 months of transgene expression when amyloid burden reached approximately 10%, we placed half of the mice on doxycycline to suppress further production of transgenic APP and human Aβ. All animals were then tested for cognitive function using the Morris water maze, radial arm water maze, and fear conditioning. Once testing was complete, we harvested the mice to examine amyloid pathology, synapse density, oligomeric Aβ, and synaptic protein levels.

## Results

Doxycycline treatment suppressed expression of transgenic APP by > 90% and diminished production of human Aβ by 70% while leaving pre-existing amyloid deposits intact. Remarkably, this treatment restored cognitive performance to the level of healthy controls in all of the behavioral tasks examined. Cognitive improvement coincided with reduced levels of synaptotoxic Aβ oligomers, greater synaptic density surrounding amyloid plaques, and increased expression of pre- and post-synaptic markers. To demonstrate that recovery was due to reduction of Aβ and not simply suppression of APP, we treated a second cohort of mice with the γ-secretase inhibitor LY411575 and observed similar although somewhat less complete recovery in both Morris water maze and synaptic markers. Finally, transgene suppression normalized activation of the actin-depolymerizing protein cofilin in a PAK-independent manner, suggesting that APP/oAβ may alter synaptic structure through the opposing Rho/ROCK pathway.

## Conclusions

These findings indicate that transient forms of APP and Aβ underlie much of the behavioral and synaptic recovery observed in this model. Our work further suggests that residual amyloid deposits need not be removed for successful intervention at this early stage of disease.

